# From memes to mobile visual language: understanding generation Z's sticker use on WeChat

**DOI:** 10.3389/fpsyg.2026.1730328

**Published:** 2026-04-20

**Authors:** Jiaqi Hu, Ribka Alan, Nurul Hidayu Mat Jusoh, Yasmin Yaccob

**Affiliations:** 1 Universiti Putra Malaysia Bintulu Sarawak Campus, Bintulu, Malaysia; 2 School of Literature and Media, Dongguan University of Technology, Dongguan, China

**Keywords:** generation Z, IPMA, NCA, UGT, WeChat stickers

## Abstract

Stickers as a defining part of internet culture have shaped how Generation Z communicates and expresses themselves in daily life. This study investigates the motivations behinds Chinese young people use stickers on WeChat, one of China's most widely used mobile platforms. Drawing on survey data from 420 young users across three Chinese representative cities from the east, west and central part of China, the findings reveal that utilitarian, social, and hedonic gratifications strongly drive sticker use, while technology factors have little influence. Using Importance-Performance Map Analysis (IPMA), Necessary Condition Analysis (NCA), and Partial Least Squares Structural Equation Modeling (PLS-SEM), the study extends Uses and Gratifications Theory (UGT) to the context of sticker usage on WeChat. By highlighting stickers as cultural tools for creative self-expression and social interaction, this research deepens the understanding of youth digital practices and contributes to broader discussions on how mobile platforms shape contemporary communication.

## 1 Introduction

Social media has transformed the way people communicate, shifting from traditional face-to-face conversations to digitally mediated interactions (Amelia and Balqis, 2023). This change is especially visible among Generation Z, born between the mid-1990s and early 2010s, who have grown up in highly connected digital environments and are often referred to as the “iGen” (Twenge, 2017). In China, WeChat plays a central role in shaping how this generation interacts. With over 1.2 billion active users, the platform integrates messaging, payments, social networking, and more, becoming a core part of young people's daily lives (Tencent, 2022). Among its many features, expressive tools such as emojis and stickers have become especially significant in everyday mobile communication (He, 2019).

Stickers, or Biaoqingbao in Chinese, have emerged as a distinctive form of visual-linguistic communication. They function not only as entertainment but also as a symbolic language system, conveying humor, irony, and subtle emotions (Zhang, 2016; Li, 2021). Unlike standard emojis, stickers are often larger, more detailed, and frequently animated. They can be used independently of text and customized to suit personal preferences (Zhou et al., 2017). For Gen Z, who tend to communicate visually, stickers enhance or even replace text, adding playfulness, ambiguity, and nuance to digital interaction (Chen and Siu, 2017).

This phenomenon reflects a wider trend on youth sub-cultural practices in China, where digital items like memes and stickers are used to express identity, creativity, and a sense of belonging (Peng, 2019). Globally, these items also play a similar role, helping young people create new ways of expressing themselves and communicating online (Milner, 2013; Shifman, 2013). Stickers, therefore, are more than communication tools. They are cultural texts that embody generational values and practices.

Cross-cultural research further shows that digital expression is shaped by sociocultural norms. Emoji use, for example, varies more by country than by language (Kejriwal et al., 2021). In collectivist cultures like China, indirect and face-saving expressions are often preferred, while individualistic cultures such as North America and Europe favor explicit communication (Cui et al., 2024). Even in digital spaces, reciprocity and etiquette continue to reflect traditional Chinese values (Liu, 2023). Although existing research has dominantly examined non-verbal visual forms as emojis and GIFs (Barbieri et al., 2016), stickers remain relatively understudied despite their prominence in Asia. Furthermore, little is known about the psychological motivations driving their adoption, particularly among Chinese Gen Z users.

This study addresses that gap by exploring Generation Z's motivations for using stickers on WeChat through the theoretical perspective of UGT. As digital technologies increasingly meet human needs ranging from basic communication to creative self-expression (Sharma et al., 2020), it is important to understand how stickers fulfill these needs. Building on prior findings, this study examines social gratifications and hedonic gratifications (Hu et al., 2023) alongside two additional factors, including technology and utilitarian gratifications. While technology gratifications are known to influence continued WeChat use (Gan and Li, 2018), their role in sticker adoption remains unclear. Furthermore, stickers offer utilitarian benefits, such as information sharing and self-presentation, enhancing personal visibility and online presence (Peng, 2019).

**Figure 1:**
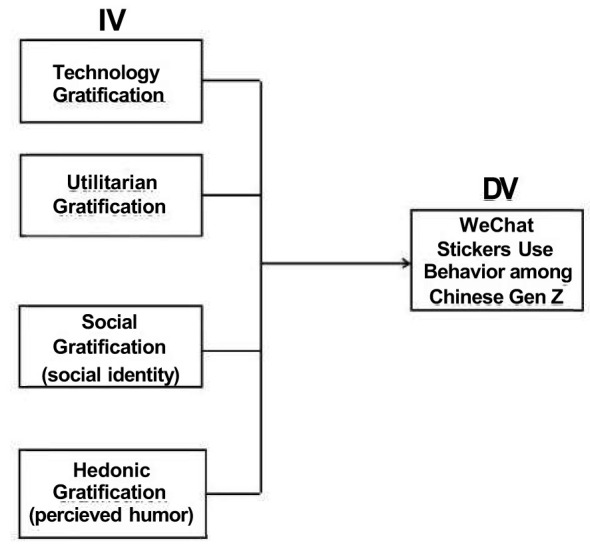
Conceptual framework.

To build on these insights, the study extends UGT by integrating multiple motivational perspectives into a unified framework. It proposes four key dimensions of gratification: technology (convenience, media appeal), utilitarian (information sharing, self-presentation), social (social presence, identity, interaction, peer influence), and hedonic (perceived entertainment, humor). The conceptual framework of this study is presented in Figure 1. To test this framework, the research employs a quantitative design using PLS-SEM, combined with IPMA and NCA to ensure robust validation.

## 2 Literature review and hypothesis

### 2.1 Emojis, stickers, and the shaping of Chinese digital subculture

Stickers, often seen as Chinese memes or internet emoticons, carry rich cultural meaning. With clear Chinese characteristics, they work as visual tools, both static and animated, that help people express emotions and ideas across social and cultural settings (Yuan and Yu, 2022).

The history of digital emoticons goes back to 1982, when Scott Fahlman introduced:-) and:-(to mark jokes and serious messages. In Japan, “Kaomoji,” or vertical emoticons, added new layers of digital expression. In China, Wang Maomao's creation of “Tuzki” in 2006, a rabbit emoticon series, marked a shift toward animated, story-like stickers (Liu and Pan, 2017).

As user-generated content grew, stickers developed into visual series with social and psychological meaning. Like Chinese internet slang, sticker language often uses humor to express negative feelings, showing the mix of irony and ambivalence in youth subculture. For example, the popular “Mouse” sticker plays on “Mouse Literature,” with phrases like “Mouse me” and “I really don't want to work anymore.” These highlight workplace fatigue, while animal-themed stickers act as metaphors for cynicism and social disappointment, using cuteness and irony to comment on reality (Sun, 2016).

As a form of visual communication, stickers carry both contradiction and resistance. Young people use them not only to express themselves but also to question mainstream ideas and affirm their identity (Zhang, 2016; Peng, 2019). Yet, despite growing interest in stickers as part of Chinese internet culture, few studies have tested the psychological motivations behind their use through quantitative models. This gap is especially clear on WeChat, where stickers are deeply embedded in youth communication but remain under-explored in research.

Studying sticker use in China provides insight into the country's evolving internet culture and the social awareness of younger generations. Meanwhile, it is important to understand why users turn to stickers from both psychological and behavioral perspectives. To address this, the present study applies UGT as its main framework, supported by related motivational theories, to model the factors driving sticker's study.

### 2.2 Gratification studies on WeChat sticker usage

Uses and Gratifications Theory has long explained how people actively choose media to meet specific needs (Katz et al., 1973). It has provided important insights into media use and has been widely applied to media platforms such as Instagram (Sheldon and Bryant, 2016), Facebook (Krause et al., 2014), Pinterest (Mull and Lee, 2014), and photo-sharing apps (Malik et al., 2016). These studies show the flexibility of UGT and its relevance in digital environments (Alhabash and Ma, 2017).

Research has also drawn attention to the communicative power of non-textual elements such as emojis and GIFs. Factors like sociability, accuracy, humor, cultural connection, and relationship maintenance are often central to their use (Chen and Siu, 2017; Church et al., 2023). These works have advanced the understanding of non-verbal cues in online communication. However, studies on stickers, especially within WeChat, remain limited, even though stickers have become a central part of everyday digital interactions in China.

Stickers act not only as expressive tools but also as a way to share emotions and strengthen social ties (Ling et al., 2021). Prior research suggests that they support fast and enjoyable communication, cross language barriers, and reflect social identity and group belonging (Jiang and Xiang, 2020; Gan, 2017). Users often report both social and hedonic gratifications from sticker use (Hu et al., 2023). Empirical studies further confirm that stickers fulfill needs such as information sharing, emotional expression, identity recognition, and entertainment (He, 2019; Kuang and Qiu, 2017).

To build on UGT, this study draws on other motivational theories to better explain the psychological drivers of sticker use. For example, Maslow's hierarchy of needs (Sharma et al., 2020) outlines a range of human needs from basic to self-actualization. Self-Determination Theory (Ryan and Deci, 2020) distinguishes between intrinsic and extrinsic motivations. Social Cognitive Theory (Bandura, 1988) stresses the importance of social influence and identity in shaping behavior. These perspectives complement UGT by showing how stickers meet different needs, namely, technology, social, utilitarian, and hedonic.

Based on these insights, this study identifies four key gratification dimensions that may influence Gen Z users' sticker practices on WeChat. It then tests a set of hypotheses through a quantitative model to examine the relative impact of each type of gratification.

#### 2.2.1 Technology gratifications in WeChat Sticker usage

Technology gratifications describe the satisfaction users gain from the features of a media platform. For example, micro-blogging offers innovative functions that enhance user experience (Liu et al., 2015). Past research has identified convenience, media appeal, and social presence as important drivers of technology-related gratifications in media use, including the internet (Papacharissi and Rubin, 2000), mobile phones (Leung and Wei, 2000), and blogging (Gulvady, 2009).

WeChat, as a multifunctional and technically innovative platform, provides several affordances such as text and voice messaging, as well as mobile social networking (Gan, 2017). Prior studies show that technology gratifications strongly influence users' intention to continue using WeChat (Gan and Li, 2018). More specifically, (Kuang and Qiu 2017) found that university students are motivated to use WeChat stickers because they are free, customized, and available in diverse categories.

Building on this, the present study defines technology gratifications as the psychological benefits Generation Z users gain from engaging with WeChat stickers. It further tests whether two first-order factors, convenience and media appeal, significantly shape Gen Z's sticker use.

H1: Technology gratifications, specifically convenience and media appeal, have a significant impact on WeChat sticker use among Chinese Gen Z.

#### 2.2.2 Utilitarian gratification in WeChat Sticker usage

Utilitarian outcomes are tied to extrinsic motivations that influence user behavior, such as quick access, information sharing, and self-documentation (Liu et al., 2015; Xu et al., 2012). In media studies, utilitarian gratifications generally refer to the efficiency and effectiveness users gain in completing tasks or social activities through media use. Prior research shows that people often turn to social media for these practical purposes, and utilitarian elements have been found to play a major role in shaping user behavior (Liu et al., 2015; Xu et al., 2012).

In the case of WeChat, (Gan and Li 2018) confirmed that utilitarian gratifications significantly affect users' intention to continue using the platform. Information sharing, in particular, has been shown to be a strong predictor of sustained engagement, as WeChat provides multiple ways to exchange content, including posts, messages, and comments.

Building on this, the present study considers how stickers also serve utilitarian functions. For younger users, stickers can support information sharing and help with self-presentation by projecting certain images of themselves in social interactions (Peng, 2019). Therefore, this study defines self-presentation and information sharing as the key utilitarian gratifications driving sticker use.

H2: Utilitarian gratifications, specifically self-presentation and information sharing, have a significant impact on WeChat sticker use among Chinese Gen Z.

#### 2.2.3 Social gratification in WeChat Sticker usage

Social gratifications are rooted in Social Cognitive Theory and arise from motivations that are met through interaction with others on media platforms (Liu et al., 2015; Gan and Li, 2018; Menon, 2022). These interactions allow users to connect with others and create meaningful social outcomes (Gan and Li, 2018). Prior research has shown that social factors such as social presence, interaction, and peer influence shape individuals' intention to use media, including online games (Li et al., 2015) and photo-sharing activities on Instagram (Menon, 2022).

In this study, social gratification is defined as the psychological rewards and satisfaction that Gen Z users gain from online interactions and relationships (Hu et al., 2023). Specifically, social presence, social interaction, peer influence, and social identity are considered key drivers of social gratification in WeChat sticker use.

H3: Social gratifications, specifically social presence, social identity, social interaction, and peer influence, have a significant impact on WeChat sticker use among Chinese Gen Z.

#### 2.2.4 Hedonic gratification in WeChat Sticker usage

Hedonic gratifications are linked to intrinsic motivations described in Self-Determination Theory (Ryan and Deci, 2020). They involve pleasure, relaxation, self-determination, and the fulfillment of socio-psychological needs through media use (Xu et al., 2012). These factors are strong predictors of social media behavior, including enjoyment, affection, and fantasy (Li et al., 2015; Xu et al., 2012).

For this study, hedonic gratification refers to the pleasurable experiences Gen Z users gain from WeChat stickers. Prior research shows that perceived entertainment and humor strongly influence sticker use (Hu et al., 2023).

H4: Hedonic gratifications, specifically perceived entertainment and perceived humor, have a significant impact on WeChat sticker use among Chinese Gen Z.

## 3 Methods

The study adapted measurement items from prior research to fit the context of WeChat sticker use. To ensure content validity, six experts in communication and psychology reviewed the items for clarity, completeness, and relevance. Based on their feedback, revisions were made, and a pilot test with 45 participants was conducted to assess reliability and understanding (Rossi et al., 2013). Minor adjustments were made following the pilot, and Cronbach's α values for all constructs were above 0.70, confirming the reliability of the final questionnaire.

To capture regional diversity, stratified sampling was employed. Participants were recruited from Dongguan, Changsha, and Chengdu, representing eastern, central, and western China. These cities were selected for their large youth populations and economic diversity, with estimated Gen Z populations of 2.62 million, 2.06 million, and 4.32 million, respectively (Hongheiku, 2023).

Following (Krejcie and Morgan 1970) guideline, a minimum sample of 384 was required. The study collected 620 responses, of which 420 were valid, including 133 from Dongguan, 103 from Changsha, and 184 from Chengdu. This exceeded the minimum sample size and met the recommended 40-observation threshold for PLS path analysis (Barclay et al., 1995).

Ethical approval was obtained from the relevant institution, with particular care given to protecting participants under 18. Participation was voluntary, incentives were provided, and data were collected using Wenjuanxin after translated from English to Chinese, a widely used online survey platform in China.

## 4 Measures

The measurement items for each construct were adapted from established scales in previous studies (Ko et al., 2005; Papacharissi and Rubin, 2000; Liu et al., 2015; James et al., 1995; Xu et al., 2012; Wang, 2017; Gan and Li, 2018). The dependent variable, WeChat sticker use, was measured with two dimensions: use intensity and use frequency. A total of seven items were adopted from prior research on social media use, including studies on SNS and Weibo (Chan et al., 2012; Chen, 2016).

For data analysis, the study employed SmartPLS 4.0 and SPSS 27.0. The PLS-SEM method was chosen because it is suitable for exploratory research and theory development (Hair et al., 2016), and performs well with formative constructs and smaller samples (Hair et al., 2011). Four gratification dimensions, technology, utilitarian, social, and hedonic, were conceptualized as second-order constructs with formative first-order indicators, and analyzed using a hierarchical component model (Wold, 1982). All items were measured on a five-point Likert scale, with 12 indicators across sub-dimensions such as convenience, media appeal, and social interaction.

## 5 Results

### 5.1 Sample characteristics

The study surveyed 420 participants, the greatest majority of whom were female and aged 15–25 years, aligning with the target Generation Z demographic. In terms of education, 75.2% were pursuing or had completed a bachelor's degree, while smaller proportions reported postgraduate or vocational qualifications. Stratified sampling ensured geographic diversity, with respondents drawn from Chengdu, Dongguan, and Changsha.

More than half of participants were students, followed by self-employed individuals. Daily WeChat usage was high, with most participants spending 2–7 h on the app and the vast majority having used it for over 6 years. Sticker use was frequent but typically brief, with most engaging for under 5 min per session. Sticker collections varied, though nearly half of respondents reported owning between 1 and 200 stickers, reflecting different levels of engagement with the feature (more detailed can be seen on Table 1).

**Table 1:** Demographic and usage profile of respondents (*n* = 420).

Measure	Items	*N*	Percentage
Gender	Male	108	25.7
	Female	312	74.3
Age	15–20	222	52.8
	21–25	136	32.4
	26–29	62	14.8
Academic qualification	High school or equivalent	17	4
	Pursuing associate's degree or vocational training	36	8.6
	Bachelor's degree student/graduate	316	75.2
	Pursuing master's degree/graduate	47	11.2
	Pursuing doctoral degree/graduate	4	1
Current location	Dongguan	133	31.7
	Changsha	103	24.5
	Chengdu	184	43.8
Employment status	Student	288	68.6
	Employed	29	6.9
	Self-employed	96	22.9
	Unemployed	7	1.7
Duration of WeChat usage	Less than 2 h/day	52	12.4
	2–4 h/day	155	36.9
	5–7 h/day	117	27.9
	8–10 h/day	52	12.4
	More than 10 h/day	44	10.5
Length of WeChat usage	Less than 6 months	0	0
	6–12 months	4	1
	2–3 years	21	5
	4–5 years	107	25.5
	6–7 years	140	33.3
	More than 8 years	148	35.2
Time duration in each session when using WeChat sticker	Less than 5 min	265	63.1
	5–30 min	105	25
	31–60 min	19	4.5
	More than 1 h	31	7.4
Amount of WeChat sticker accumulation	1–200	196	46.7
	201–400	120	28.6
	401–600	33	7.9
	More than 600	71	16.9

### 5.2 Measurement model

The measurement model was assessed separately for formative and reflective constructs. For formative constructs, validity was evaluated using item weights and loadings (Cenfetelli and Bassellier, 2009). All outer weights exceeded 0.2, and loadings were above 0.7 (Table 2), confirming statistical significance.

**Table 2:** Item weights and loadings of formative second-order constructs.

Constructs	Item	Weights	*t* value	Loadings	Variable inflation factor
Technology gratification	Convenience	0.529	82.958	0.923	2.046
	Media appeal	0.551	54.725	0.929	2.046
Utilitarian gratification	Information sharing	0.561	61.081	0.907	1.675
	Self-presentation	0.545	67.412	0.901	1.675
Social gratification	Social presence	0.273	50.492	0.872	2.676
	Social identity	0.284	65.521	0.905	3.259
	Social interaction	0.300	56.961	0.917	3.405
	Peer influence	0.272	57.027	0.843	2.237
Hedonic gratification	Perceived entertainment	0.520	133.144	0.957	3.296
	Perceived humor	0.524	117.431	0.958	3.296

Reliability was checked through multicollinearity tests, with VIF values ranging from 1.675 to 3.405, well below the recommended threshold of 10 (Table 2). These results indicate that all four formative constructs met the required criteria (Hair et al., 1998; Mathieson et al., 2001).

For reflective constructs, reliability was examined using Cronbach's α and composite reliability (CR), both of which were above the recommended 0.70 threshold, confirming internal consistency (Nunnally, 1978; Chin, 1998). The result showed that all reflective constructs satisfy the required standards, with CR ranging from 0.814 to 0.929 and Cronbach's α values between 0.802 and 0.929.

Convergent validity was established with CR values above 0.80, AVE values between 0.645 and 0.832, and item loadings exceeding 0.707, after removing three low-loading items from self-presentation, peer influence, and perceived entertainment (Table 3).

**Table 3:** Convergent validity (after deleted items).

Constructs	Item loading	Composite reliability (rho-a)	AVE	Cronbach's α
Convenience (CON)		0.883	0.809	0.882
CON1	0.906			
CON2	0.881			
CON3	0.912			
Media appeal (MA)		0.897	0.698	0.892
MA1	0.868			
MA2	0.838			
MA3	0.877			
MA4	0.820			
MA5	0.772			
Information sharing (IS)		0.920	0.804	0.919
IS1	0.870			
IS2	0.910			
IS3	0.908			
IS4	0.898			
Self-presentation (SP)		0.899	0.831	0.897
SP2	0.855			
SP3	0.941			
SP4	0.937			
Social presence (SOP)		0.917	0.749	0.916
SOP1	0.863			
SOP2	0.804			
SOP3	0.879			
SOP4	0.903			
SOP5	0.876			
Social identity (SID)		0.874	0.723	0.871
SID1	0.891			
SID2	0.899			
SID3	0.827			
SID4	0.779			
Social interaction (SI)		0.892	0.748	0.885
SI1	0.917			
SI2	0.920			
SI3	0.844			
SI4	0.768			
Peer influence (PI)		0.825	0.645	0.817
PI1	0.771			
PI2	0.762			
PI4	0.829			
PI5	0.846			
Perceived entertainment (PE)		0.901	0.832	0.899
PE1	0.908			
PE2	0.883			
PE3	0.922			
Perceived humor (PH)		0.929	0.824	0.929
PH1	0.905			
PH2	0.916			
PH3	0.913			
PH4	0.897			
WeChat sticker use intensity (WSI Int)		0.814	0.729	0.813
WSI Int 1	0.829			
WSI Int 2	0.882			
WSI Int 3	0.849			
Frequency of WeChat sticker use (WSI Freq.)		0.825	0.652	0.821
WSI Freq 1	0.804			
WSI Freq 2	0.869			
WSI Freq 3	0.732			
WSI Freq 4	0.820			

## 6 Discussion

### 6.1 Research findings

The research sought to investigate the overall goal of examining the impact of four gratification on WeChat sticker usage among Chinese Generation Z. Specifically, three research hypothesis were proposed, with the corresponding findings summarized below.

**Figure 2:**
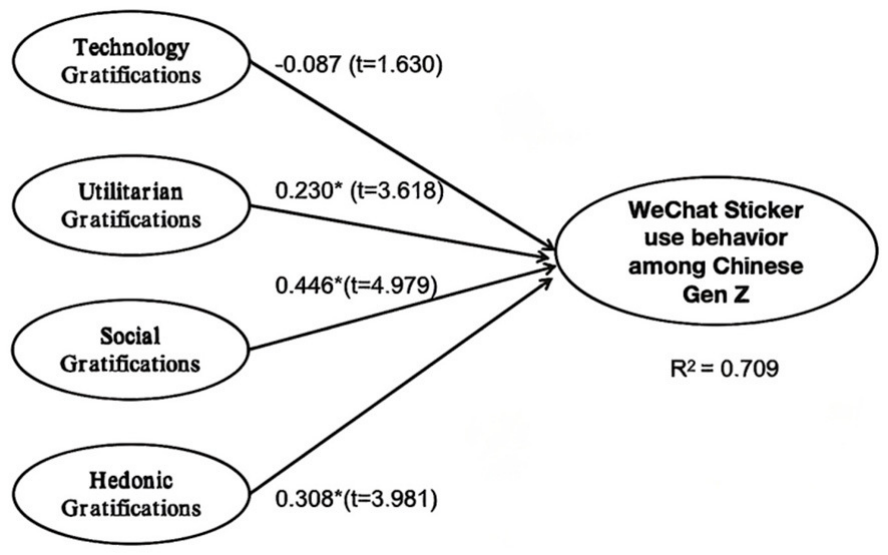
Result of PLS.

**Figure 3:**
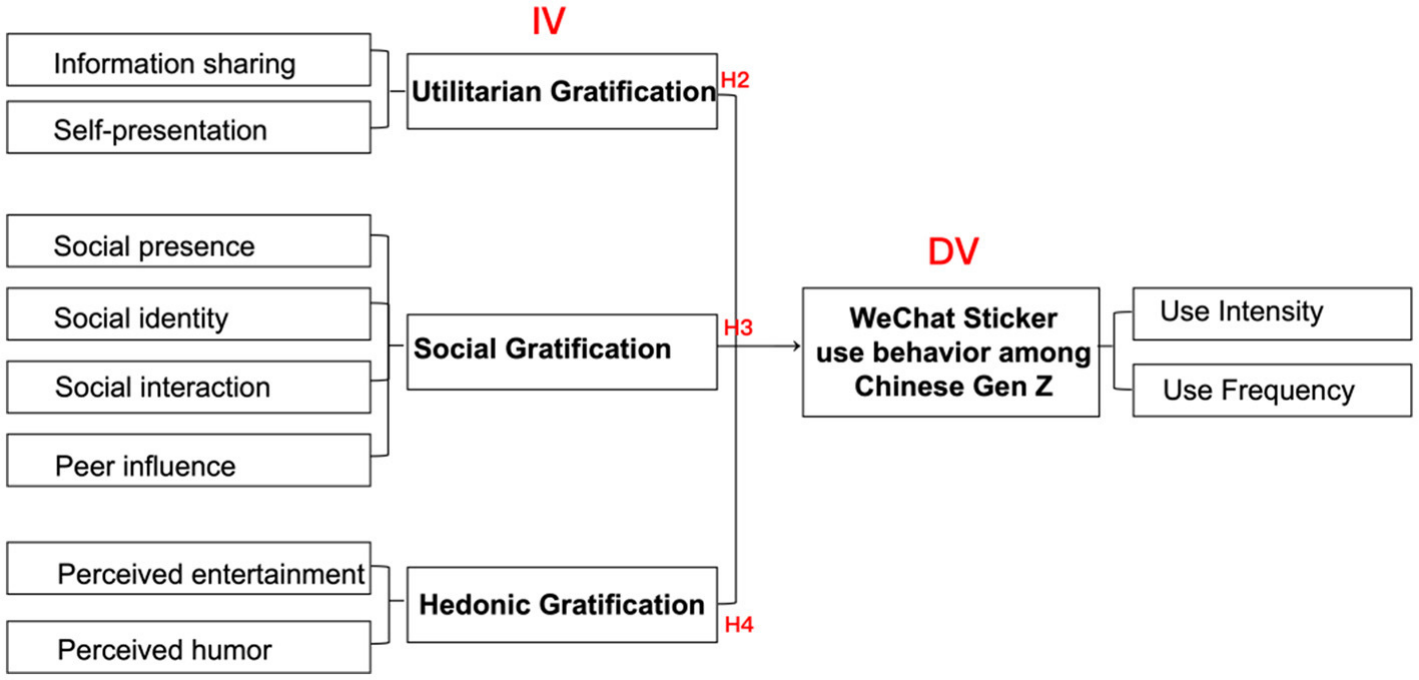
Results of structural model-research hypotheses.

As shown in Figure 2, the PLS-SEM results revealed that utilitarian, social, and hedonic gratifications had a statistically significant impact on sticker usage. In contrast, technology gratification did not show a significant effect. Accordingly, hypotheses H2, H3, and H4 received support, while H1 was rejected (see from Figure 3 and Table 4).

**Table 4:** Results of structural model-research hypotheses significant at ^**^p ≤ 0.01, ^*^p < 0.05.

H	Relationship	Path	*t*-value	*p*-value	Direction	Decision
H1	TG -> WSI	−0.089	1.630	0.103	Negative	Not supported
H2	UG -> WSI	0.230	3.618	0	Positive	Supported^**^
H3	SG -> WSI	0.446	4.979	0	Positive	Supported^**^
H4	HG -> WSI	0.308	3.981	0	Positive	Supported^**^

Together, the four gratification factors explained 70.9% of the variance in WeChat sticker usage, indicating a high level of predictive accuracy. Therefore, the findings provide strong support for the study. The result aligns with previous research demonstrating the direct influence of social, hedonic, and utilitarian gratifications on social media usage (Li et al., 2015; Malik et al., 2016; Gan and Li, 2018; Menon, 2022). However, contrary to earlier finding (Kuang and Qiu, 2017), no significant effect of technology gratification was observed in this study.

To test the effects of utilitarian, social, and hedonic gratifications, a hierarchical analysis across four models was conducted. Along with assessing the *R*^2^ values for all endogenous constructs, the alterations in *R*^2^ when a particular exogenous construct is excluded were analyzed to determine the *f*^2^ effect size. This method assesses if the excluded construct significantly influences the endogenous variables (as illustrated in Table 5).

**Table 5:** Summary of PLS results.

Independent variables	Dependent variable: WeChat sticker use behavior among Chinese Gen Z			
	Model 1	Model 2	Model 3	Model 4
Technology gratifications	−0.050	−0.043	0.009	−0.089
Utilitarian gratifications		0.483	0.21	0.230
Social gratifications	0.622		0.629	0.446
Hedonic gratifications	0.297	0.454		0.308
*R* ^2^	0.693	0.667	0.672	0.709
Δ*R*^2^	0.691	0.664	0.670	0.706
*f*^2^-statistic	0.062	0.144	0.127	

Model 1 comprises technology gratifications, social gratifications, and hedonic gratifications. Model 2 incorporates technology gratifications, utilitarian gratifications, and hedonic gratifications. Model 3 encompasses technology gratifications, utilitarian gratifications, and social gratifications. In model 3, four different gratifications are included.

The findings indicate that utilitarian gratifications increase the *R*^2^ from 69.3% to 70.9% (a rise of 1.6%, and *f*^2^ = 0.062), demonstrating a small effect size; social gratifications boost the *R*^2^ from 66.7% to 70.9% (a rise of 4.2%, and *f*^2^ = 0.144), reflecting a medium effect size; additionally, hedonic gratifications raise the *R*^2^ from 67.2% to 70.9% (an increase of 3.7%, and *f*^2^ = 0.127), also signifying a medium effect size.^1^ The result aligns with previous studies indicating that social and hedonic gratifications significantly influence WeChat sticker usage, consistent with earlier findings identifying them as strong motivators for media use (Li et al., 2015).

In PLS analysis, *Q*^2^ is a measure of predictive relevance that evaluates the model's capability to forecast the values of the dependent variable using the independent variables (Geisser, 1974; Stone, 1974). Table 6 reveals that all *Q*^2^ values surpass 0.35, demonstrating substantial predictive relevance of the model. The values for the four gratifications span from 0.771 to 0.917, with the *Q*^2^ for WeChat Sticker usage behavior in Chinese Gen Z achieving 0.622.

**Table 6:** Results of PLS.

Path Relationship	Original sample (O)	Standard deviation (STDEV)	T statistics (|O/STDEV|)	*P* values	*F* ^2^	VIF	*Q* ^2^
TG -> WSI	−0.089	0.053	1.630	0.103	0.012	2.231	0.622
UG -> WSI	0.230	0.064	3.618	0	0.052	3.514	
SG -> WSI	0.446	0.09	4.979	0	0.144	4.749	
HG -> WSI	0.308	0.077	3.981	0	0.125	2.607	
WSI -> WSI Int	0.943	0.006	158.686	0	0.089	1	0.645
WSI -> WSI Freq	0.94	0.007	140.752	0	0.736	1	0.573
CON -> TG	0.528	0.007	78.983	0		2.05	0.850
MA -> TG	0.551	0.01	53.751	0		2.05	
IS -> UG	0.62	0.012	53.864	0		1.506	0.785
SP -> UG	0.553	0.008	68.99	0		1.506	
SOP-> SG	0.275	0.005	50.962	0		2.665	0.771
SID-> SG	0.285	0.004	63.717	0		3.222	
SI -> SG	0.302	0.005	55.026	0		3.545	
PI -> SG	0.272	0.005	55.476	0		2.191	
PE -> HG	0.517	0.004	134.127	0		3.465	0.917
PH -> HG	0.525	0.005	113.35	0		3.465	

### 6.2 Importance-performance matrix analysis (IPMA) and necessary condition analysis (NCA)

**Figure 4:**
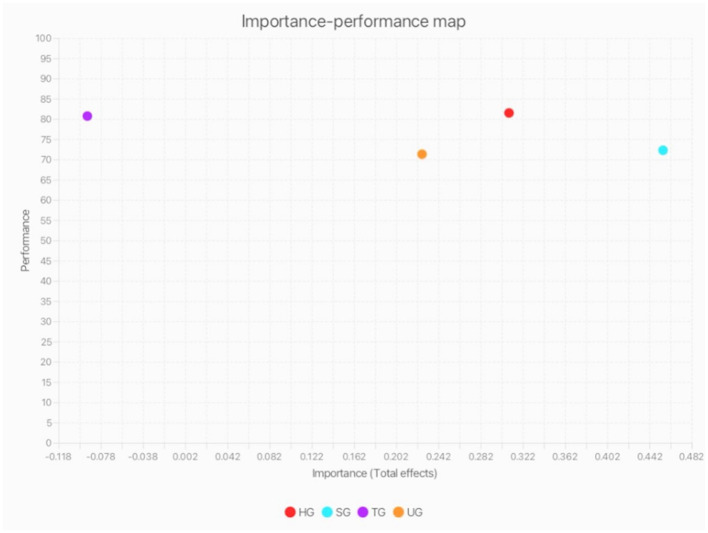
IPMA for gratification obtained from WeChat sticker use model.

The IPMA builds on the PLS results by evaluating not only the impact of each factor but also how well each performs in practice. While PLS identifies the strength of relationships through path coefficients, IPMA adds performance scores to give a comprehensive understanding (Slack, 1994). As shown in Table 7, social gratification has the highest importance (0.455) but lower performance (72.26) compared to hedonic gratification, which performs better (81.49). The matrix in Figure 4 shows that hedonic, social, and utilitarian gratifications are the most influential in driving sticker use, while technology gratification, though rated highly in performance, ranks low in importance, suggesting it is not a key motivator.

**Table 7:** Table of importance-performance map analysis (IPMA).

Construct	Importance	Performance
HG	0.309	81.491
SG	0.455	72.262
TG	−0.091	80.718
UG	0.226	71.299

IPMA helps identify influential but underperforming factors, pointing to areas for practical improvement. NCA complements this by identifying which factors are essential for the outcome to occur (Dul, 2016; Bergh et al., 2022). As seen in Table 8, hedonic and utilitarian gratifications are shown to significantly impact sticker usage, with hedonic gratification having the strongest effect (0.475). Social gratification, although important in the PLS results (0.446), was not found to be necessary in the NCA, suggesting it may play a supporting or indirect role. Technology gratification was not significant in either analysis. These results highlight hedonic and utilitarian motivations as the main drivers of WeChat sticker use among Gen Z.

**Table 8:** Total effects from the PLS-SEM analysis and necessity effect sizes (CE-FDH ceiling line).

Construct	WeChat sticker	
	Total effect (PLS)	CE-FDH effect size (NCA)
HG	0.308^***^	0.475^***^
SG	0.446^***^	0.127
TG	−0.089	0.030
UG	0.230^***^	0.216^***^

^*^p <0.05; ^**^p <0.01; ^***^p <0.001.

### 6.3 Sticker-based communication and user wellbeing

Instead of identifying the key gratification drivers of sticker use, the present findings carry important implications for understanding how sticker-based communication relates to users' psychological and social wellbeing.

The emergence of hedonic gratification as the strongest predictor of sticker use (*f*^2^ = 0.127) points to its significant role in emotions and psychological wellbeing. Humor and entertainment as the core dimensions of hedonic gratification are well-established mechanisms for managing negative affect, alleviating daily stress, and cultivating positive emotional states (Li et al., 2015; Xu et al., 2012). For Chinese Gen Z users operating under considerable academic, occupational, and social pressures (Wang et al., 2023), stickers function as low-cost, readily accessible emotional coping tools. The use of self-deprecating memes or humorous animal characters, for instance, allows users to lighten psychological burdens through shared levity. This hedonic dimension aligns closely with the affective component of subjective wellbeing (Diener, 1984), where the experience of positive emotions through everyday interactions, including digital ones, constitutes a foundational element of individual flourishing. Furthermore, within the framework of Self-Determination Theory (Ryan and Deci, 2000), the intrinsic enjoyment derived from expressive sticker use satisfies the need for autonomy and competence, both of which are recognized antecedents of psychological wellbeing. Platform designers and mental health practitioners should therefore recognize that hedonic features embedded in messaging applications are not only serving for entertainment affordances but meaningful contributors to users' emotional health.

Besides, the significant influence of social gratification (*f*^2^ = 0.144), which is the largest effect size among all significant predictors, underscoring the centrality of interpersonal connection in sticker use, with direct implications for social wellbeing. (Keyes 1998) conceptualized social wellbeing as encompassing dimensions of social integration, social acceptance, social contribution, social actualization and social coherence, all of which are reinforced through the communicative practices stickers facilitate. By enabling the expression of empathy, humor, solidarity, and shared cultural references without the constraints of verbal articulation, stickers lower the emotional labor of communication and enhance relational quality (Kuang and Qiu, 2017). In a collectivist cultural context such as China's, where maintaining relational harmony (guanxi) and face-saving communication are paramount (Liu, 2023), stickers serve as cultural signals that sustain group cohesion and affirm belonging. This aligns with Keyes' (1998) notion of social integration, wherein individuals experience wellbeing through their sense of shared community and mutual recognition. Conversely, the underperformance of social gratification identified by IPMA (performance score: 72.26) suggests that despite its motivational contribution, users may not yet feel fully satisfied in their social connectedness through sticker use. This gap presents both a wellbeing concern and an opportunity that platforms should enhance features that deepen reciprocal emotional expression and facilitate richer relational bonding, thereby translating high motivational importance into genuine social wellbeing outcomes.

Although utilitarian gratifications demonstrated a smaller effect size (*f*^2^ = 0.062), their role in self-presentation and information sharing carries notable implications for identity-related dimensions of wellbeing. Psychological wellbeing, has been regarded as intra-personal attributes related to adaptation, self-actualization, and empowerment (Garcia, 2012), all of which are implicated in how individuals manage their digital self-presentation. For Gen Z users, strategically selecting stickers that project their desired identities, whether intellectual, humorous, or culturally aware, constitutes a form of active identity work that reinforces self-awareness and contributes to a sense of authentic self-expression (Peng, 2019). When such self-presentation is socially validated through peer interaction, it further supports self-esteem and self-acceptance (Valkenburg et al., 2021). In this sense, the utilitarian function of stickers extends beyond pragmatic efficiency, it serves as a medium through which users negotiate and affirm their sense of self within networked communities.

In general, these findings suggest that the three gratification dimensions, hedonic, social, and utilitarian, can led to interrelated wellbeing outcomes, including emotional regulation and positive affect, social connectedness and belonging, and identity affirmation and self-acceptance. This supports the view active use of social media can improve subjective wellbeing, thereby creating stimulating feelings of social connectedness (Verduyn et al., 2017). Importantly, however, these benefits are contingent on the quality of engagement. Passive or heavy sticker use may paradoxically undermine wellbeing (Twenge and Campbell, 2019). This dual nature highlights the need for digital literacy education that helps users engage with expressive communication tools in intentional, emotionally authentic ways.

## 7 Conclusion

This study examined the key motivations behind WeChat sticker use among Chinese Gen Z users through the theoretical framework of UGT. The findings reveal that utilitarian, social, and hedonic gratifications significantly influence sticker use, whereas technology gratification, despite its assumed importance in prior studies, shows no direct effect. One possible explanation lies in the unique digital upbringing of Gen Z. Having grown up in a media-saturated environment, Gen Z are highly proficient in digital technologies and regard them as natural extensions of daily life rather than sources of novelty or satisfaction (Cilliers, 2017; Francis and Hoeful, 2018; Dimock, 2019). For this concern, the technological affordances of social media platforms are taken for granted, functioning as background conditions that enable interaction rather than motivate it. In this stance, technology gratifications may have become less salient, while utilitarian, social and hedonic needs take precedence. This aligns with research suggesting that technological features often act as contextual or moderating variables rather than primary motivators of media use (Sundar and Limperos, 2013; Kejriwal et al., 2021).

A major contribution of this study lies in identifying social gratifications, particularly social presence, interaction, identity, and peer influence, as strong predictors of WeChat sticker use. The introduction of social identity as a motivational dimension extends the UGT framework and aligns with Social Identity Theory (Tajfel, 1978), emphasizing how users engage with media to reinforce group belonging. This is particularly meaningful within China's collectivist culture, where communication emphasizes relational harmony, subtle emotional expression, and the maintenance of social ties (Cui et al., 2024; Liu, 2023). Stickers thus serve as semiotic tools that express cultural values and sustain social cohesion within peer networks.

From a cultural standpoint, stickers are not merely instruments of entertainment but represent a visual language through which young users construct and negotiate identity. Common themes, such as self-deprecating humor or animal-based memes, reflect everyday frustrations, identity struggles, and workplace fatigue, symbolizing a form of “micro-resistance” through humor and irony (Wang et al., 2023). This creative use of visual expression demonstrates how collectivist values and social constraints are subtly challenged or reaffirmed within digital spaces.

Utilitarian gratifications, such as information sharing and self-presentation, also play a notable role, supporting prior research that highlights the practical and expressive functions of visual tools in online communication (Xu et al., 2012; Peng, 2019). Hedonic gratifications, especially perceived humor and entertainment, emerged as the strongest predictors of sticker use, improving model accuracy by 3.7%. This reflects Gen Z's preference for emotionally engaging and socially resonant content, where humor strengthens peer connections and enhances platform attachment (Barta et al., 2023).

Finally, with over 81% of respondents reporting frequent sticker use, primarily for sharing rather than creating, this pattern may indicate a cultural inclination toward communal participation rather than individual production, reflecting both collectivist norms and personality traits that value conformity and social approval (Liu and Sun, 2020).

Critically, this study contributes to an emerging understanding of how sticker-based visual communication intersects with users' psychological and social wellbeing. The three significant gratification dimensions identified in this study, including hedonic, social, and utilitarian, collectively map onto distinct wellbeing outcomes. Hedonic gratifications, rooted in humor and entertainment, facilitate emotional regulation and positive affect, functioning as accessible tools for everyday stress relief and mood enhancement (Diener, 1984). Social gratifications, encompassing interaction, peer influence, and social identity, sustain relational bonds and foster a sense of belonging. They are key constituents of social wellbeing as conceptualized by (Keyes 1998). Utilitarian gratifications, particularly self-presentation, support identity enhancement and self-acceptance, contributing to psychological wellbeing through authentic digital self-expression (Garcia, 2012). These findings collectively suggest that, for Chinese Gen Z, sticker use is not simply a communicative act but a form of digital emotional labor that carries meaningful implications for how individuals manage their emotional lives, maintain social connections, and affirm their sense of self within networked communities. Platforms and developers should therefore consider these wellbeing dimensions when designing expressive communication features, recognizing its emotional and social functions that can meaningfully contribute to users' flourishing rather than serving merely as engagement metrics. Concurrently, promoting digital literacy that encourages authentic sticker use may help mitigate potential wellbeing risks associated with passive or anxiety-driven use patterns.

Theoretically, this study extends the UGT by integrating complementary motivational perspectives, notably Self-Determination Theory and Social Identity Theory. Traditionally, UGT has emphasized individual cognitive and affective needs, such as information seeking and entertainment, while paying limited attention to intrinsic or extrinsic motivation dynamics and collective identity formation. By incorporating motivational theories, this study broadens UGT's explanatory power from an individual-centered framework to one that also accounts for social and cultural dimensions of media engagement. Specifically, the integration of Self-Determination Theory clarifies the internal mechanisms behind user satisfaction, distinguishing intrinsic motivations (hedonic enjoyment, utilitarian usefulness) from extrinsic motivations (social recognition, peer influence). Meanwhile, applying Social Identity Theory situates sticker use within users' identity practices, highlighting how digital communication reinforces belonging and group cohesion, which is especially salient in collectivist cultural contexts.

This theoretical implication allows UGT to better explain contemporary media behavior, where emotional expression, identity recognition, and social connection are often intertwined. By recognizing technology's role as an enabling rather than determining factor, the model posits technological affordances as contextual conditions that shape, but do not drive, user motivation. Through this lens, UGT evolves into a more dynamic framework capable of capturing the complex interplay between psychological needs, social identity, and technological environments in digital media use.

Furthermore, the application of PLS-SEM, IPMA, and NCA empirically substantiates this multidimensional structure, enhancing the theory's analytical robustness and offering methodological support for future studies on interactive media behavior.

Practically, the findings suggest that mobile platforms aiming to engage Gen Z should focus on relational, expressive, and emotionally resonant features, rather than just technical innovation. Stickers, as emotionally and culturally embedded digital tools, serve not only communicative functions but also shape identity, humor, and social interaction, thereby actively contributing to users' psychological and social wellbeing. Designers and platform policymakers should thus approach expressive communication features as wellbeing resources with digital guidance, rather than regarded it as auxiliary entertainment affordances.

This study also presents limitations. The gender imbalance within the sample may have influenced the findings, and the quantitative design limited deeper exploration of cultural dynamics. Future research should consider examining gender-specific patterns and cross-cultural differences in sticker use. Qualitative approaches, such as interviews or ethnographic methods, could offer richer insights into the symbolic, emotional, and social meanings of stickers within digital subcultures. Extending the UGT framework to incorporate cultural and affective dimensions may enhance our understanding of how mobile visual communication functions within diverse sociocultural contexts.

## Data Availability

The original contributions presented in the study are included in the article/supplementary material, further inquiries can be directed to the corresponding author.

## Footnotes

1 Cohen's f^2^-statistic = (R^2^AB – R^2^A)/(1– R^2^AB), where R^2^A is the variance accounted for by a set of one or more independent variable A, and R^2^AB is the combined variance accounted for by A and another set of one or more independent variables B. f^2^ of 0.02, 0.15, and 0.35 are termed small, medium, and large effect sizes, respectively.
